# PVP/aprepitant microcapsules produced by supercritical antisolvent process

**DOI:** 10.1038/s41598-024-60323-z

**Published:** 2024-05-09

**Authors:** Zhuo Zhang, Guizhou Hao, Xuemei Sun, Feibo Wang, Dengbo Zhang, Dedong Hu

**Affiliations:** 1 College of Mechanical and Vehicle Engineering, Linyi University, Linyi, 276000 China; 2 Center for New Drug Pharmacological Research of Lunan Pharmaceutical Group, State Key Laboratory of Generic Manufacture Technology of Chinese Traditional Medicine, Linyi, 273400 China; 3 College of Electromechanical Engineering, Qingdao University of Science and Technology, Qingdao, 266061 China

**Keywords:** Supercritical antisolvent process, Coaxial annular nozzle, Industrialization, Microcapsules, Aprepitant, Chemical engineering, Nanoscale materials

## Abstract

The supercritical antisolvent (SAS) process was a green alternative to improve the low bioavailability of insoluble drugs. However, it is difficult for SAS process to industrialize with limited production capacity. A coaxial annular nozzle was used to prepare the microcapsules of aprepitant (APR) and polyvinylpyrrolidone (PVP) by SAS with N, N-Dimethylformamide (DMF) as solvent. Meanwhile, the effects of polymer/drug ratio, operating pressure, operating temperature and overall concentration on particles morphology, mean particle diameter and size distribution were analyzed. Microcapsules with mean diameters ranging from 2.04 μm and 9.84 μm were successfully produced. The morphology, particle size, thermal behavior, crystallinity, drug content, drug dissolution and residual amount of DMF of samples were analyzed. The results revealed that the APR drug dissolution of the microcapsules by SAS process was faster than the unprocessed APR. Furthermore, the drug powder collected every hour is in the kilogram level, verifying the possibility to scale up the production of pharmaceuticals employing the SAS process from an industrial point of view.

## Introduction

For the sake of improving the dissolution rate of insoluble drugs and maintaining their properties, particle size reduction is a widely used method in the pharmaceutical industry since it can increase the surface area to volume ratio and increase the specific surface area in contact with the solvent. The supercritical fluid (SCF) technology has been extensively proposed as an alternative in micronization for its merits of easy control operating parameters, low or no residual solvent, and relatively low temperatures. In particular, supercritical antisolvent (SAS) process is the most widely used method with smaller particles size and narrow particle size distribution^1^. In this process, the precipitation mechanism is governed by mass transfer kinetics, fluid dynamics and high-pressure equilibria as well as the kinetics of nucleation and growth of particles^2,3^. Supercritical CO_2_ (SC-CO_2_) is the best candidate SCF for its advantages of economic, non-toxic, non-flammable and easily-accessible critical point (*P*_*C*_ = 7.38 MPa, *T*_*C*_ = 31.13 ℃)^4,5^.

Nevertheless, it is difficult for SAS process to meet the industrial demand of microparticles since it is still in the laboratory stage for the equipment manufacturing. The nozzle, as the key part of crystallization by SAS process, plays a crucial role in enhancing the dispersion of the solution, thereby affecting the size and morphology of the particles. The traditional nozzles used for SAS process were capillary nozzles^6^, laser drilling nozzles^7^, sintered plate nozzles^8^ and ultrasonic enhanced nozzles^9^. However, these nozzle outlets are all micropores with diameters ranging from tens to hundreds of micrometers, which have problems such as small flow area, easy blockage, low efficiency, and cannot meet the industrial production demand of particles preparation. It has to be pointed out that the coaxial annular nozzle provides the potential for the industrial production of nanoparticles by SAS since the annular gap area is greatly increased while the nozzle annular size remains unchanged. At the same time, under the same optimum size of 100 μm^10–13^, the cross-sectional area of the annular structure of the nozzle is nearly 10^3^ times larger than that of the traditional circular structure, which can improve the production of particles.

**Figure 1:**
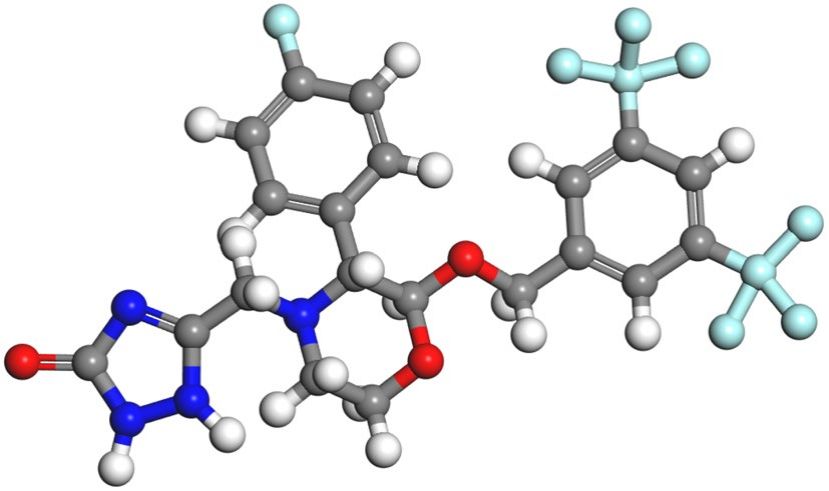
Molecular structure of ARP.

Aprepitant (APR), a white powder with a molecular weight of 534.43, is a powerful and effective antiemetic agent to inhibit vomiting and nausea caused by cancer chemotherapy. The molecular structure of ARP is shown in Fig. 1. It is the first Neurokinin-1 (NK1) antagonist approved by the Food and Drug Administration (FDA) in 2003. APR is categorized as the Biopharmaceutics Classification System (BCS) class IV drug with low solubility and low permeability. Additionally, it is rarely soluble in isopropyl acetate and ethanol, slightly soluble in acetonitrile and insoluble in water^14–18^. To our knowledge until now, some drugs can be coprecipitate with hydrophilic polymeric carriers to form microspheres to improve the dissolution rate of insoluble drugs. Nonetheless, it is hard to acquire microcapsules using SAS, as the drug and polymer have the tendency to precipitate separately, especially when nanoparticles are obtained by homogeneous nucleation and growth. Indeed, irregular and coalescing particles with broad particle size distribution^19^ and low encapsulation efficiency^20,21^ were prepared and, in many cases, the explanation of microcapsules was problematic^22–24^. In other cases, some successful SAS coprecipitated powders were reported using different active principles and polymers. Polyvinylpyrrolidone (PVP) is a widely used water-soluble synthetic biopolymer in the preparation of drug–polymer composite particles. It is an inactive ingredient included in FDA-approved drug products, which possesses a superiority to control the crystallization to form spherical microparticles, and the drug is well dispersed in the PVP network. Many investigations have proven that PVP was successfully precipitated into micro and nanoparticles employing SAS^25–31^.

Considering the limitations of traditional techniques in micronizing ARP, in the present work, PVP/ARP microcapsules using the coaxial annular nozzle by SAS was put forward, aiming to prepare microparticles that can improve the drug dissolution of APR in an industrial scale. The effect of polymer/drug ratio, operating pressure (P), operating temperature (T) and overall solute concentration (C_tot_) on particle morphology and particle size were analyzed and discussed. Proper characterization methods were performed to confirm the success of microcapsules.

## Materials, methods and procedures

### Materials

Polyvinylpyrrolidone (PVP, average molecular weight 10 kg/mol), aprepitant (ARP, purity 99.9%), nitrogen (purity 99.9%) and CO_2_ (purity 99.9%) were supplied by Lunan Houpu Pharmaceutical Co., LTD (China). N, N-Dimethylformamide (DMF, purity 99.5%) and ethanol (purity 100%) were provided by Xilong Scientific Co., Ltd. (China). Sodium dodecyl sulfate (SDS, purity 99.9%) purchased from Suzhou KuangShi Chemical Co. Ltd (China) was selected as the dissolution medium. Solubility tests performed at room temperature showed that the solubilities of ARP and PVP in DMF were about 420 mg/mL and 400 mg/mL, respectively.

### SAS apparatus and procedure

**Figure 2:**
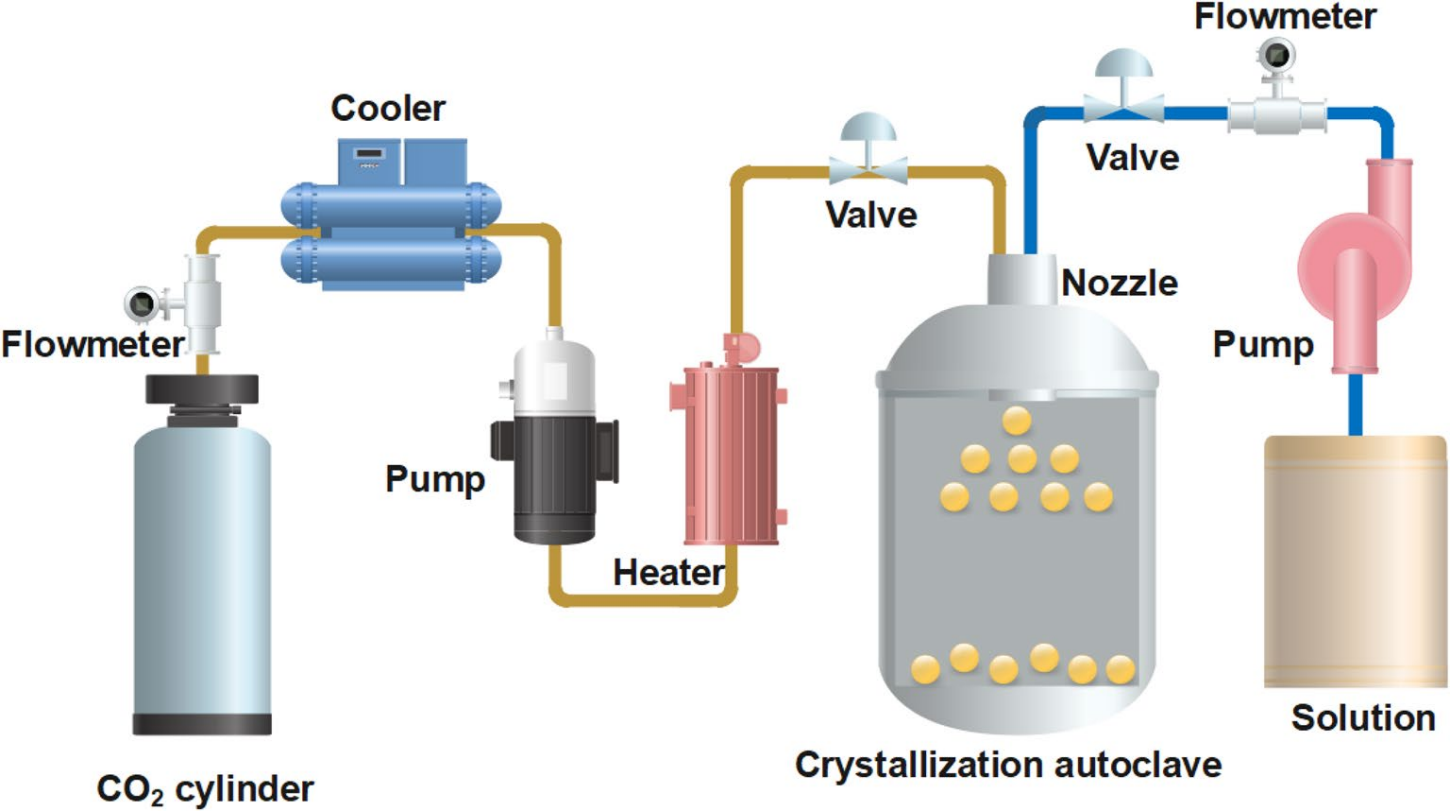
Schematic of the detailed experimental device.

The schematic diagram of handmade SAS equipment is shown in Fig. 2. A plunger pump (model 3 TB-50/50, China) equipped with a refrigeration equipment (model ACW-100BH-02, China) for the pumping head, is used to convey liquid carbon dioxide. An advection pump (model 2 J-XZ, China) delivers the mixture solution. A crystallization autoclave of 5L volume, equipped with a 100-μm-coaxial annular nozzle on the top, is used as the precipitation chamber. A porous metallic frit of 5 μm diameter located at the bottom of the crystallization autoclave is adopted to collect the precipitated powders and allows CO_2_-solvent-solution to pass through. The pipelines and coaxial annular nozzle are wrapped with insulating materials to ensure that the temperature is in the pregnant stage. A second collection chamber downstream of the microvalve is used to recover liquid solvent. The buffer autoclave and crystallization autoclave are heated using thermostatic water baths, and the accuracy of temperature sensors are ± 0.1 ℃.

Firstly, CO_2_ in the cylinder is cooled down to the dense liquid phase by the refrigeration equipment and then pumped to the stainless-steel buffer autoclave. Further, the buffer autoclaves and coaxial annular nozzle are heated until the desired conditions are reached. Then the liquid solution (containing DMF, ARP, PVP and SDS) is sprayed into the crystallization autoclave and contacted with SC-CO_2_ in the coaxial annular nozzle, resulting in the solute supersaturated and precipitated. After the aimed amount of solution is delivered, SC-CO_2_ continues to flow for at least 60 min to remove the residual solvent altogether. If the final purge with SC-CO_2_ is not performed, the solvent may condense during the depressurization step and solubilize or modify the precipitates. Finally, venting the CO_2_ slowly until the precipitator depressurized down to atmospheric pressure and the precipitated samples are then collected on a metal filter with pore diameters of 0.1 μm at the bottom of the crystallizer autoclave. All SAS experiments were carried out using a CO_2_ flow rate of 250 L/min, a solution flow rate of 1 mL/min and DMF as the liquid solvent.

### Analytical methods

Scanning electron microscopy (SEM) was performed using an EM-30 Plus (COXEM, Inc., Korea) to observe the surface morphology of samples. Sample powders were placed on a double-sided adhesive carbon tape, which was mounted on an aluminium pin stub. Gold was coated in vacuum for 3 min by sputtering coater and observed by SEM at 15 kV voltage.

Laser Particle Size Analyzer (Master size 3000, Malvinpanaco, England.) was employed to calculate the particle size and overall distribution. The dispersion pressure is 2.0 bar, the vibration injection rate is 40%, and the particle refractive index is 1.564.

The thermal behaviour of samples was performed by a Differential Scanning Calorimeter (DSC 200 F3, Germany) using the Mettler stare system. The powder samples were accurately weighed (5 ± 0.5 mg), placed into an aluminium pan and heated from 30 to 300 °C at 10 °C/min under a nitrogen purge of 50 ml/min. Each analysis was performed in duplicate.

X-ray diffractograms (XRD) were obtained by an X-ray powder diffractometer (Empyrean, PANalytical B.V., Netherlands). The analysis was carried out with Cu Kα radiation at 40 mA and 40 kV with a step size of 0.013° and scan step times of 18.87 s, covering a 2θ range of 3–50°.

The drug content of SAS processed powders was analysed by High Performance Liquid Chromatography (HPLC, Dionex UltiMate 3000, Dionex, USA). The elution was obtained using a C18 column (Inertsil ODS-3, 50 mm × 4.6 mm, 5 μm) with a flow rate of 1.5 ml/min and injection volume equal to 20 μL. The detection wavelength is 210 nm. The mobile phase utilized was consists of potassium dihydrogen phosphate solution (3.40 g dissolved in 900 ml water, pH adjusted to 3.0 with phosphoric acid, and diluted to 1000 ml with water) and acetonitrile (55:45). The drug loading of the particles was calculated by the following Eq. (1):

1 $$\% {\text{ Drug loading }} = \, \left( {{\text{mass of loading drug}}/{\text{ total mass of the particles}}} \right) \times {1}00\%$$

Drug dissolution studies were performed using the Intelligent dissolution tester RCY-1400 T (Tianjin, China) and Shimadzu ultraviolet spectrophotometer UV-2401PC (USP, Hangzhou Coulomb Technology Co. LTD, China). 10 mg of each unprocessed ARP and PVP/ARP sample were added to different dissolution vessels containing 900 ml of 0.8% SDS. The temperature of the dissolution medium was maintained at 37 ± 0.5 °C and the paddle device speed was 50r/min. At certain time intervals of 5, 10, 15, 30, 60, 90 and 120 min, 5 ml sample was withdrawn using a 0.45 μm syringe filter and analysed spectrophotometrically at 264 nm. Each sample was repeated thrice and fresh medium in the same quantity was added to the vessel to maintain constant volume. The formula is as follows:

2 $$\% {\text{ Drug release }} = \, \left( {{\text{Sample absorbance}}/{\text{ Standard absorbance}}} \right) \times {1}00\%$$

where the standard was 100% of drug release.

The residual amount of DMF in the SAS processed powders were carried out in an Agilent 7890 A gas chromatograph equipped with flame ionization detector (GC-FID). DMF was accurately weighed and dissolved in ethanol to prepare a control solution with a concentration of about 0.089 mg/ml. Similarly, samples were accurately weighed and dissolved in ethanol to prepare the test solution with a concentration of about 100 mg/ml. The capillary column (model DB-WAX, Agilent, USA) was connected to the detector, 30 mm length, 0.53 mm i.d., 1.0 μm film thickness. The injector was maintained at 200 ℃ (split mode, ratio 10:1), and nitrogen was used as the carrier gas (3.0 mL/min). The oven temperature was programmed from 50℃ at a rate of 20 ℃/min to 200 ℃. The chromatographic method starts with an initial temperature at 50 ℃ for 2 min, then increased to 200 ℃ at 20 ℃/min and maintained at 200 ℃ for 10 min.

## Results and discussion

A list of the performed experiments was reported in Table 1, where the obtained morphology, mean diameter (m.d.) and standard deviation (s.d.) were shown. All the employed experimental conditions were performed above the Mixture Critical Point (MCP) of DMF/CO_2_ binary system based on the *P-x–y* phase equilibrium reported in literature^32^.

**Table 1:** Summary of SAS experiments performed on PVP/ARP.

Runs	PVP/ARP (w/w)	P (MPa)	T (℃)	C_tot_ (mg/ml)	Morphology	m.d. (μm)	s.d. (μm)
1	0:1	12	40	50	MP	2.04	2.7482
2	1:0	12	40	50	MP	9.44	13.3438
3	1:3	12	40	50	MP	2.53	1.6969
4	1:1	12	40	50	MP	2.91	2.4110
5	3:1	12	40	50	MP	3.73	2.3326
6	6:1	12	40	50	MP	4.64	3.1660
7	9:1	12	40	50	MP	9.84	21.6170
8	6:1	9	40	50	MP	7.49	7.5089
9	6:1	15	40	50	MP	2.35	1.8309
10	3:1	12	35	50	MP	2.78	2.2671
11	3:1	12	50	50	Liq	–	–
12	3:1	12	40	100	MP	3.90	2.5092
13	3:1	12	40	150	MP	4.27	2.6598

*MP* microparticles, *Liq* liquid.

**Figure 3:**
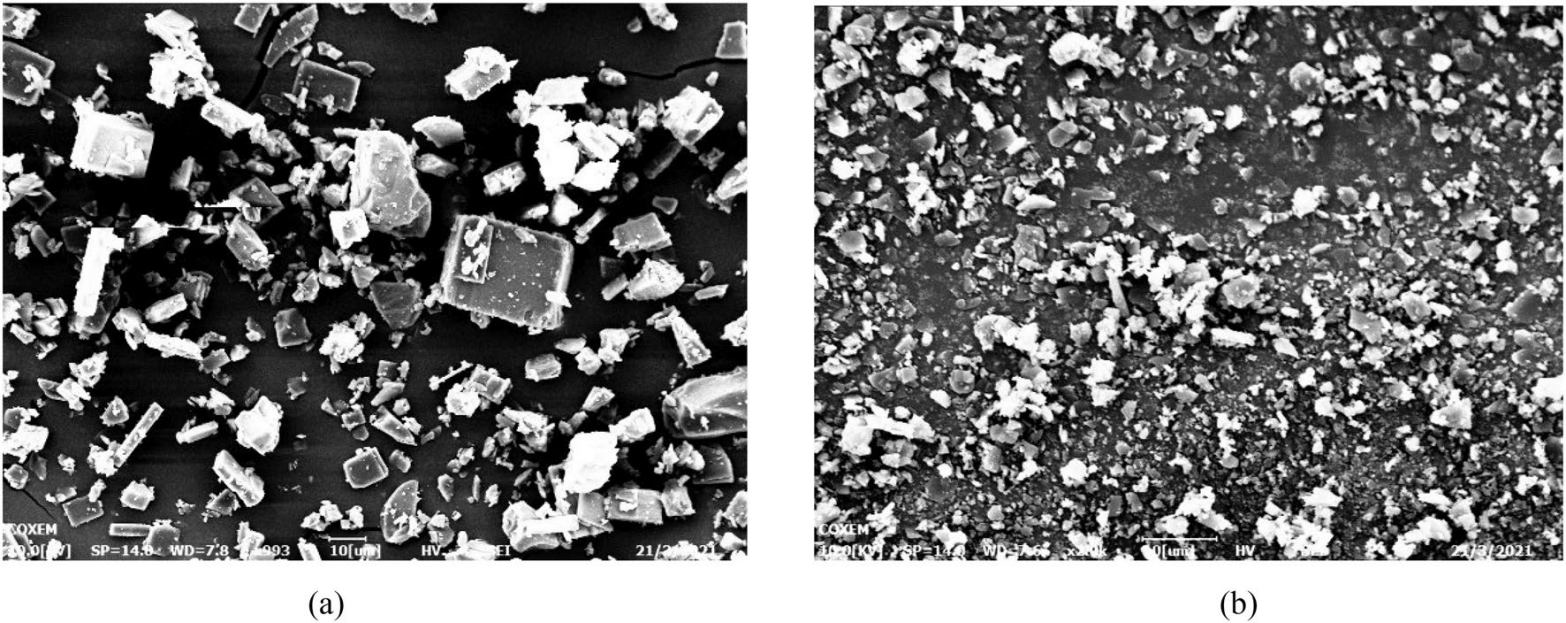
SEM images of ARP, (**a**) Original ARP and (**b**) ARP particles precipitated from DMF solution at 12 MPa, 40 ℃ and 50 mg/ml.

**Figure 4:**
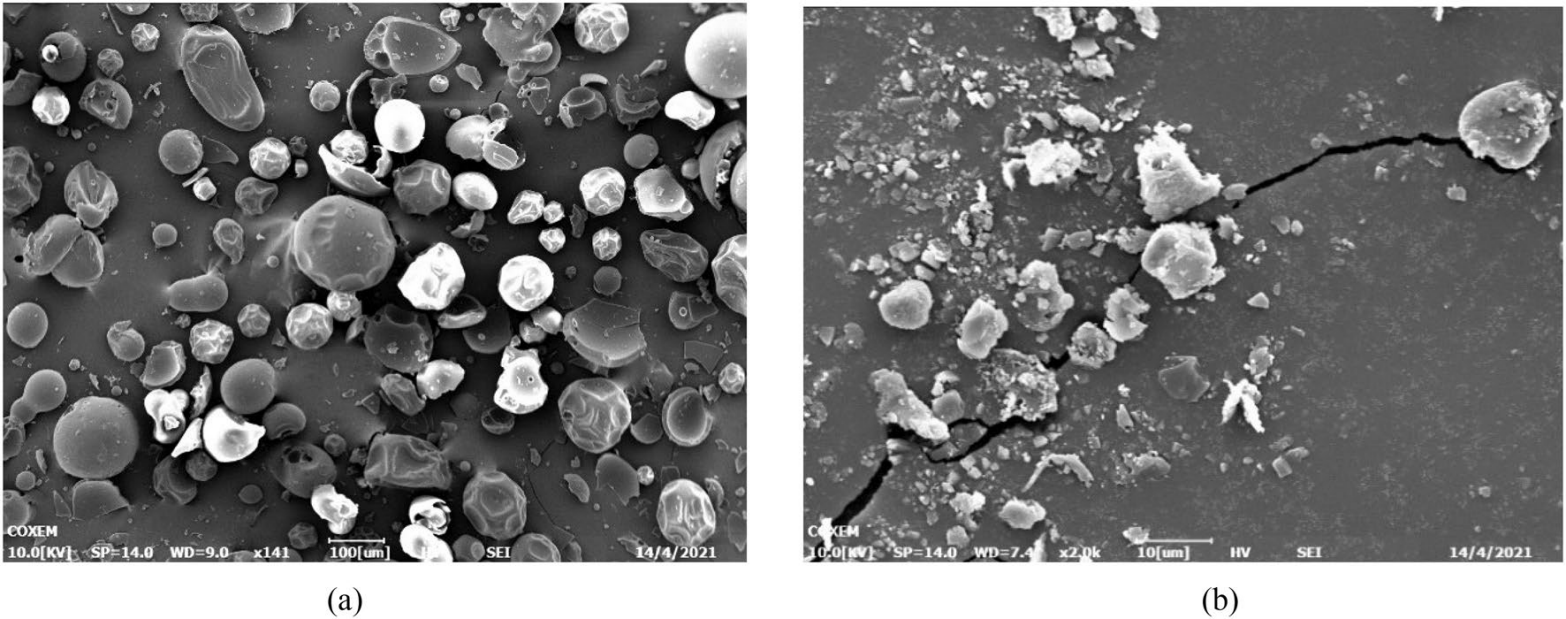
SEM images of PVP, (**a**) Original PVP and (**b**) PVP particles precipitated from DMF solution at 12 MPa, 40 ℃ and 50 mg/ml.

Some preliminary experiments to process ARP and PVP separately were necessary to observe their precipitation behavior when processed by SAS. The first set of experiments was carried out to prepare ARP alone at 12 MPa, 40 ℃ and at an overall solute concentration of 50 mg/ml (run 1 in Table 1). It is observed that SAS processing altered the morphology and particle size significantly. Original ARP comprises strip-like particles (Fig. 3a), whereas, the powders obtained by SAS processing exhibits irregular plate-like particles with an average particle size of 2.04 μm (Fig. 3b). Then PVP was precipitated at the same operating conditions alone (run 2). Original PVP has spherical and large particles while PVP processed by SAS precipitated in the form of irregular-shaped and large particles, as reported in Fig. 4.

The observation of the SEM images for ARP and PVP indicates that the drug and the polymer precipitate with completely different morphology, even when processed at the same operating conditions, from a SAS point of view. Several experiments were attempted with the systems PVP/ARP to verify that it is suitable for microcapsules.

### Effect of PVP/drug ratio

**Figure 5:**
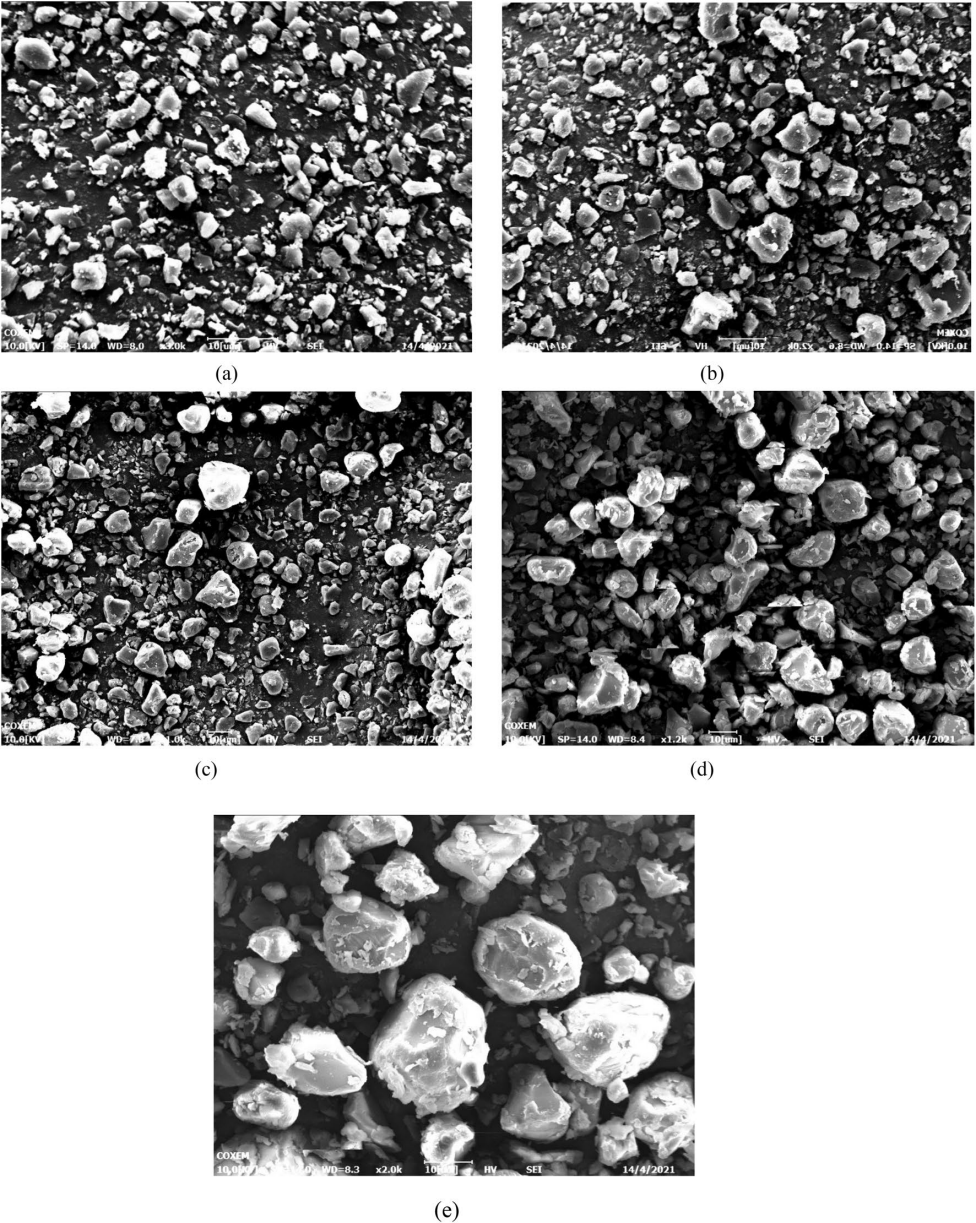
SEM images of PVP/ARP microcapsules at 12 MPa, 40 °C and 50 mg/ml at different PVP/ARP ratios: (**a**) 1:3, (**b**) 1:1, (**c**) 3:1, (**d**) 6:1 and (**e**) 9:1.

The first set of coprecipitate experiments was investigated at 12 MPa, 40 °C and 50 mg/ml, varying the PVP/ARP ratio from 1:3 to 9:1. When PVP/ARP 1:3 ratio (run 3) was processed, crystals and massive particles with an average diameter equal to 2.53 μm were obtained, as observed in Fig. 5a. Increasing the PVP/ARP ratio at 1:1 (run 4) and 3:1 (run 5), crystals and sub-micro particles with average diameters equal to about 2.91 μm and 3.73 μm were prepared, as shown in Fig. 5b and c. Subsequently, the ratio PVP/ARP were increased at 6:1 and 9:1 (runs 6 and 7), well-separated microparticles were obtained, as it is possible to observe in Fig. 5d,e.

**Figure 6:**
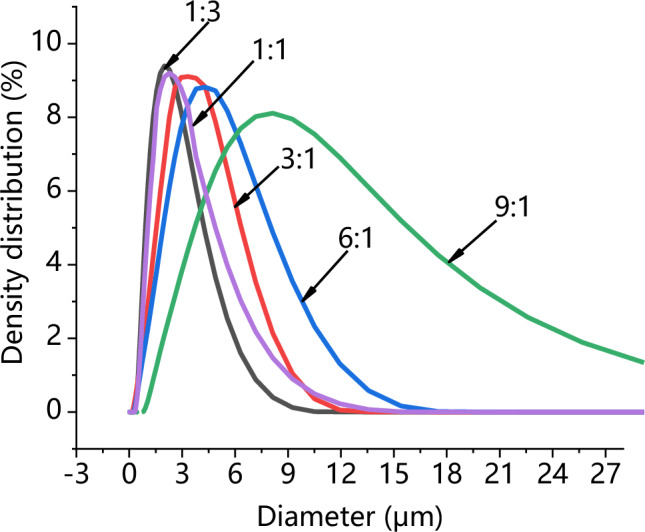
Particle size analyses of PVP/ARP microcapsules at 12 MPa, 40 °C and 50 mg/ml at different PVP/ARP ratios.

Comparing the particle size distribution (PSD) of PVP/ARP particles precipitated at different PVP/ARP ratios, obtained in this set of experiments, it was observed that, increasing the percentage of polymer in the injected solution, the mean size of the particles increased and the PSD enlarged, as shown in Fig. 6.

### Effect of operating pressure

**Figure 7:**
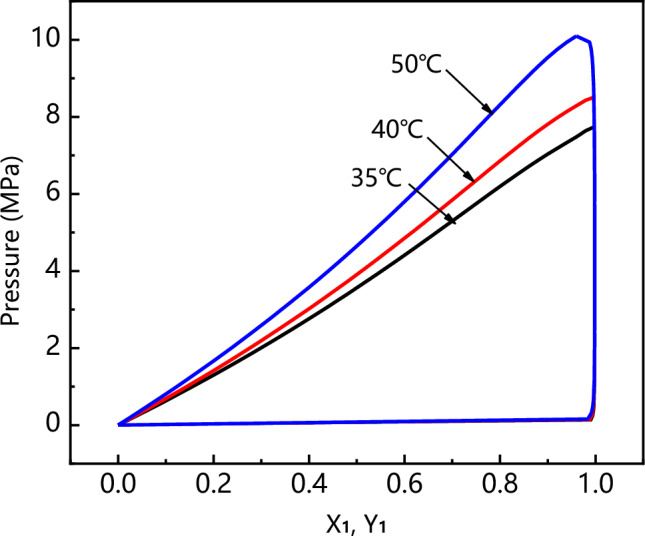
The *P-x-y* phase equilibrium of CO_2_-DMF mixtures at different temperatures calculated by PR equation of state.

**Figure 8:**
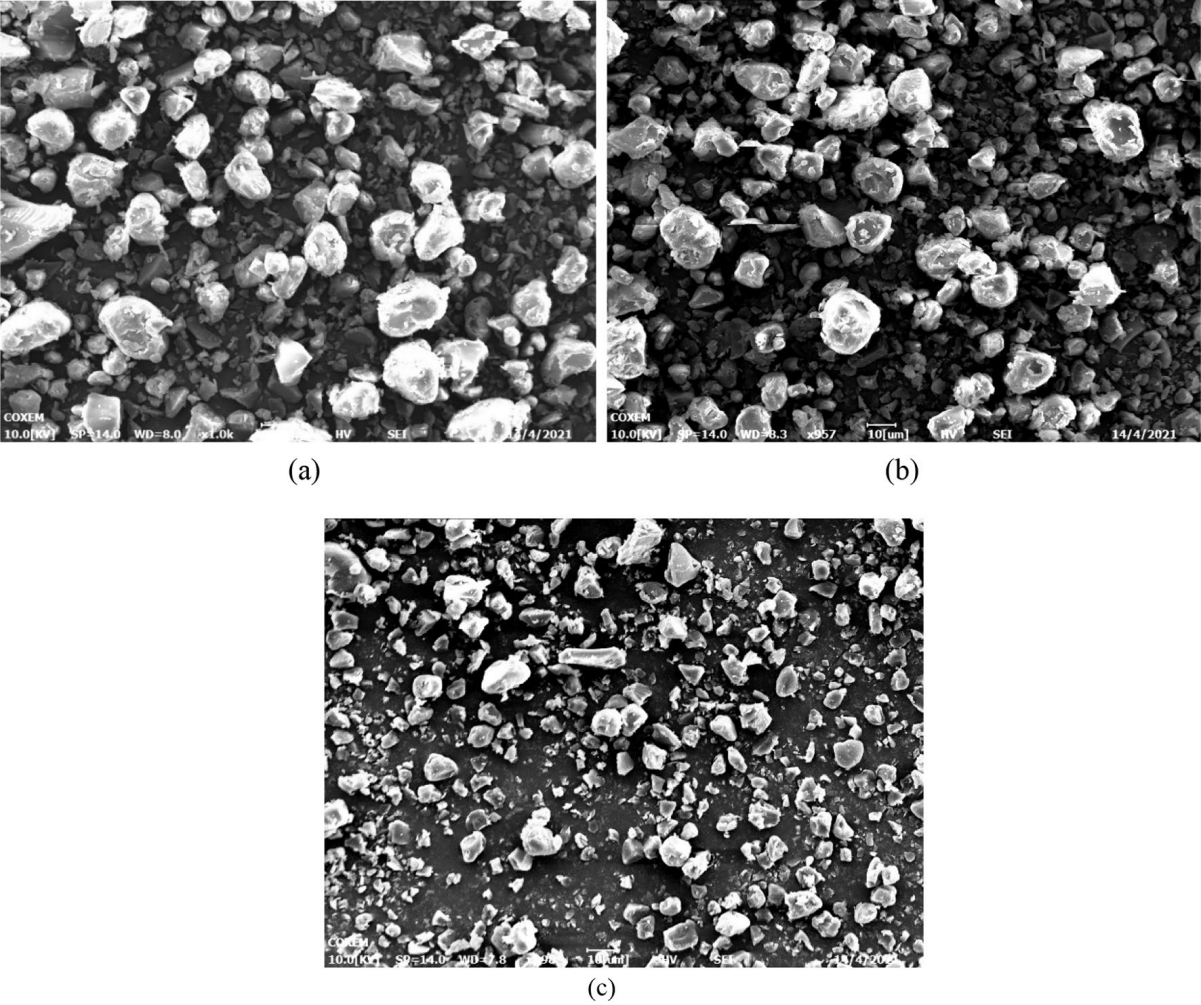
SEM images of PVP/ARP microcapsules at 3:1, 40 °C and 50 mg/ml at different pressures: (**a**) 9 MPa, (**b**) 12 MPa and (**c**) 15 MPa.

**Figure 9:**
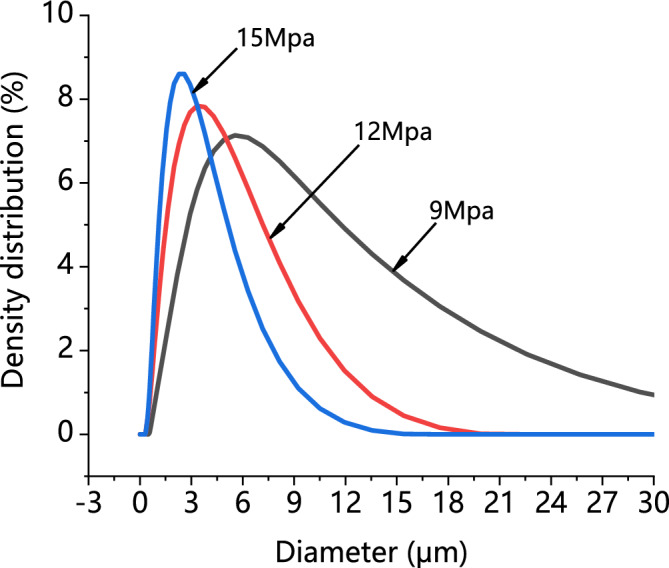
Particle size analyses of PVP/ARP microcapsules at 6:1, 40 °C and 50 mg/ml at different pressures.

In order to evaluate the influence of the SAS operating pressure, three experiments (runs 6, 8 and 9 in Table 1) were analyzed, i.e., theoretically near above and far above the MCP of the CO_2_/DMF system. It is seen from Fig. 7 that the MCP of the DMF/CO_2_ binary system was 8.4 MPa at 40 ℃. The temperature was fixed at 40 °C and overall solute concentration was kept at 50 mg/min, varying the operating pressure at 9 MPa, 12 MPa and 15 MPa, respectively. Regarding the PVP/ARP system, working at the pressure of 9 MPa (run 8) and 12 MPa (run 6), spherical microparticles with a mean diameter of 7.49 μm and 4.64 μm were obtained, as shown in Fig. 8a and b. Increasing the pressure to 15 MPa (run 9), smaller spherical microparticles with a mean diameter of 2.35 μm were still produced, but a few particles were irregular, as shown in Fig. 8c. Comparing the PSD of the precipitated powders, it was possible to note that as the pressure increases, the average diameter decreases and PSD shrink, as shown in the density distribution curve in Fig. 9.

### Effect of operating temperature

**Figure 10:**
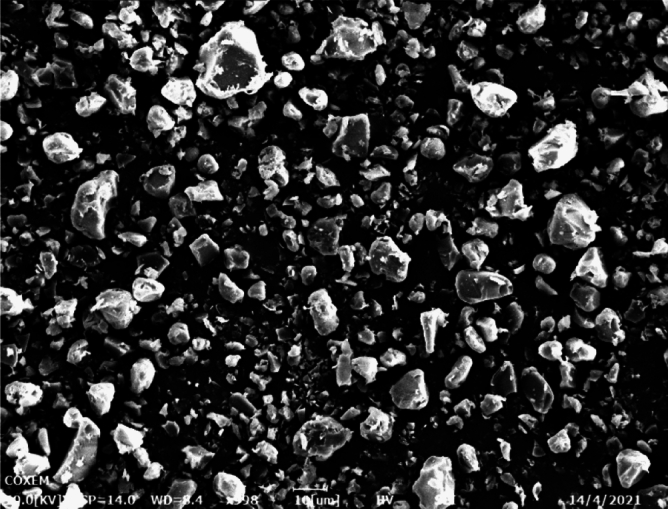
SEM images of PVP/ARP microcapsules at 12 MPa, 3:1, 40 °C and 50 mg/ml.

**Figure 11:**
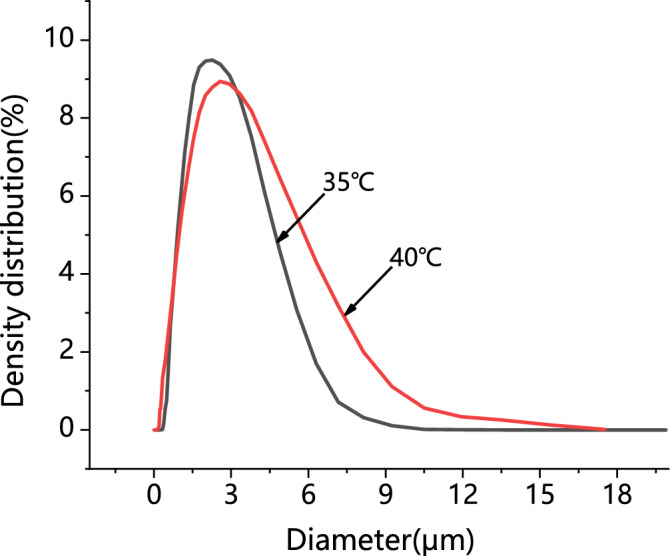
Particle size analyses of PVP/ARP microcapsules at 3:1, 12 MPa and 50 mg/ml at different temperatures.

To study of the operating temperature effect, experiments are performed with three levels, 35 ℃, 40 ℃ and 50 ℃, while other parameters were maintained at a fixed level. Regarding the PVP/ARP system, by selecting a PVP/ARP ratio of 3:1, well-separated microparticles with a mean diameter of 2.78 μm and 3.73 μm were obtained respectively when processing at the temperature of 35 °C and 40 °C (runs 10 and 5), as shown in Figs. 10 and 11. Whereas, a certain amount of liquid was found inside the precipitation chamber at the condition of run 11. It was considered an unsuccessful experiment since the presence of PVP and APRT modified high-pressure vapor–liquid equilibria (VLEs) of the binary system CO_2_/DMF and enlarged the miscibility hole, resulting in the operating point located inside the two-phase region^33^. Therefore, no further tests were carried out at 50 °C and the subsequent experiments with the PVP/ARP system were performed at 40 °C. It can be observed that, increasing the operating temperature, the average particle size increased and the PSD enlarged, as it is possible to observed in Fig. 10.

### Effect of the overall solute concentration

**Figure 12:**
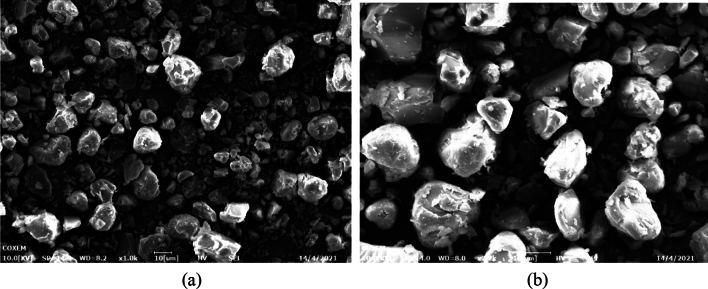
SEM images of PVP/ARP microcapsules at different overall solute concentration: (**a**) 50 mg/m, and (**b**) 150 mg/m.

**Figure 13:**
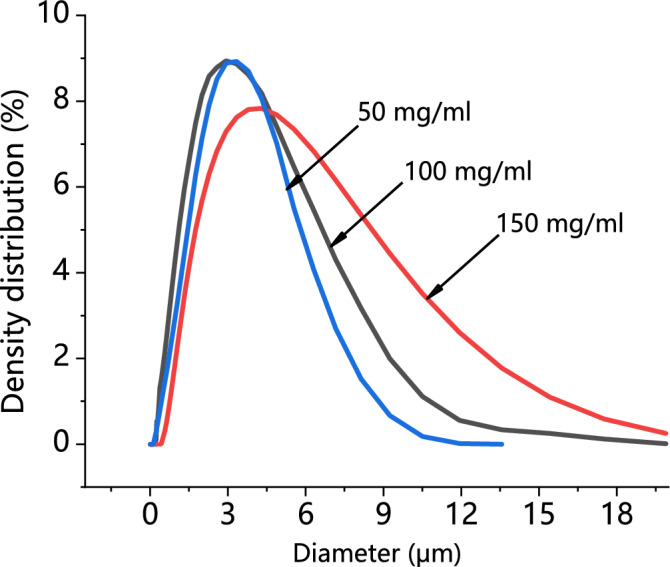
Particle size analyses of PVP/ARP microcapsules at 12 MPa, 3:1, and 313 K at different overall solute concentrations.

The effect of the overall solute concentration of solutes dissolved in DMF was evaluated using three concentrations: 50 mg/ml, 100 mg/ml and 150 mg/ml (runs 5, 12 and 13); in this set of experiments, the chosen pressure and temperature were fixed at 12 MPa and 40 ℃, whereas the PVP/ARP ratio was 3:1. Microparticles with an average diameter of about 3.73 μm were obtained under the concentrations of 50 mg/ml (run 5), as reported in Fig. 12a. Increasing the concentration at 150 mg/ml (run 13), microparticles were still produced with a mean diameter of about 4.27 μm, as it is possible to observe in Fig. 12b. It was observed from Fig. 13 that increasing the overall solute concentration, the mean particle sizes increased slightly with wider PSD; this result is consistent with some other experimental conclusions of SAS precipitate reported in the literature^34,35^.

### Characterization of precipitates

**Figure 14:**
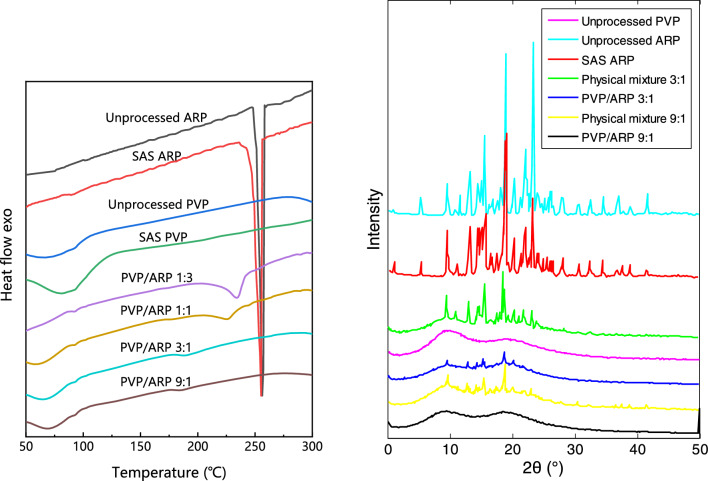
Unprocessed and SAS processed samples using the different mixtures; (**a**) DSC thermograms; (**b**) XRD analysis.

DSC was performed on unprocessed ARP and PVP, SAS processed drugs and polymer and PVP/ARP microcapsules at different ratios, to analyze the changes in the thermal transition of the drug and the polymer in the microcapsules. The DSC thermograms are reported in Fig. 14a. The unprocessed APR and SAS processed APR show narrow endothermic peaks at about 256.4 ℃ and 245.9 ℃ (melting point), respectively. It is probably ascribable to the reduction in particle size of the SAS processed APR or the change in the crystalline structure. A broad endothermic peak ranging from 50 °C to 100 °C related to the loss of volatile components were observed both in unprocessed PVP and SAS processed PVP thermograms. Both PVPs before and after SAS process showed amorphous structures. SAS processed PVP/ARP coprecipitate at different ratios show both the endothermic peaks. However, with the increase of PVP ratio, the peak of the drug is remarkably reduced in its intensity (results not shown) and the ARP characteristic peaks were slightly shifted to lower temperatures. This behavior is due primarily to the solvent effect of PVP. In any case, DSC analyses confirmed the amorphous characteristic of SAS processed PVP/ARP coprecipitate.

The crystallization behavior of unprocessed PVP, unprocessed ARP, SAS processed PVP, SAS processed ARP, physical mixture PVP/ARP and SAS processed PVP/ARP coprecipitate at different ratios were evaluated by XRD. As indicated in Fig. 14b, the unprocessed ARP shows the typical crystalline structure; meanwhile, SAS processed ARP exhibits the same spectral pattern, but with lower intensity. The physical mixture PVP/ARP (3:1 and 9:1) is less crystalline than unprocessed ARP significantly since the peak intensity decreased, which is attributed to the presence of amorphous polymer. However, the unprocessed PVP and SAS processed PVP/ARP microcapsules (3:1 and 9:1) present the amorphous structure. This may be due to the fact that ARP molecules were embedded in the amorphous PVPs when microcapsules were formed, thus, the diffraction peaks of the drugs disappeared. This result can also be confirmed by considering the DSC analyses.

**Figure 15:**
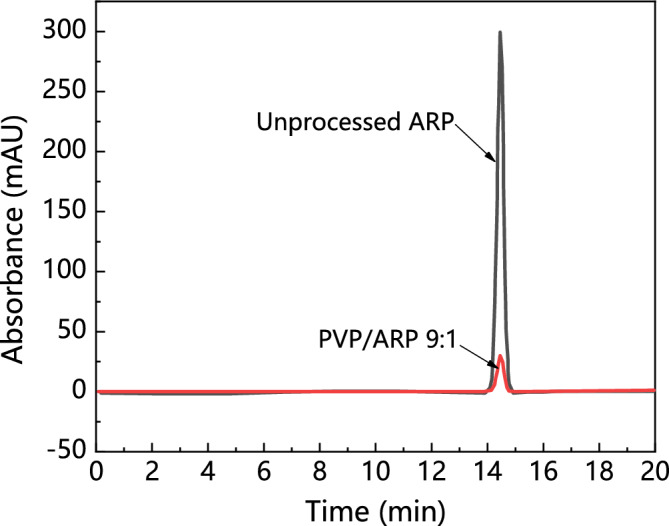
HPLC measurements of unprocessed ARP and SAS processed sample.

Drug loading analyses were carried out for the powders obtained at 12 MPa, 40 ℃, 50 mg/ml solution concentration and polymer/drug at different polymer/drug ratios. Figure 15 exhibits the HPLC analyse of unprocessed ARP and SAS processed sample. It was observed from Fig. 15 that there was only one peak for the unprocessed ARP with a retention time of 14.468 min and a peak area of 88.2103 mAU*min, whereas, PVP/ARP 9:1 have one peak with a retention time of 14.461 min and a peak area of 9.1253 mAU*min. Similarly, other PVP/ARP microcapsules with the polymer/drug ratios of 1:1, 3:1, 6:1 and 9:1 have the peak area of 90.055 mAU*min, 27.0481 mAU*min, 13.5903 mAU*min and 9.1253 mAU*min, respectively. Thus, the drug contents of samples were calculated as 50.59%, 23.00%, 13.04% and 9.15% for the polymer/drug ratios of 1:1, 3:1, 6:1 and 9:1. It is revealed that both the solutes existed in the samples.

**Figure 16:**
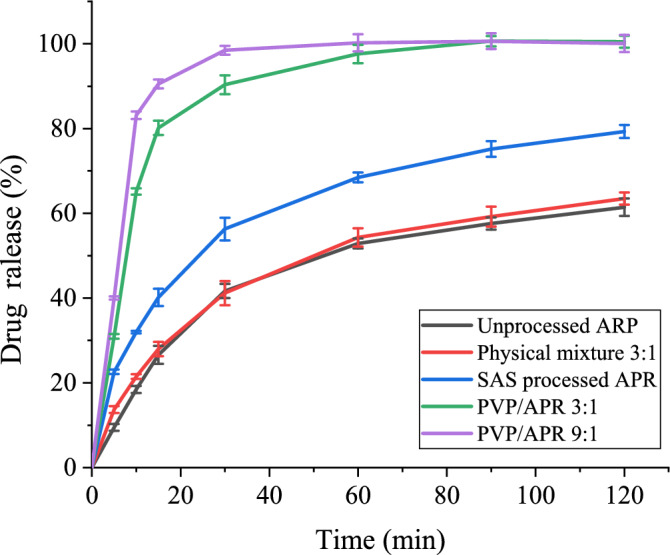
Dissolution profiles of APR in 1% SDS at 12 MPa, 40 ℃ and 50 mg/ml.

Drug dissolution studies were performed using the intelligent dissolution tester and analyzed using the UV–vis spectrophotometer to compare the release kinetics of different samples and, therefore, to verify the possible faster release of the microcapsules. The drug release of each sample in 1% SDS was monitored for 2 h and the percentage of dissolved APR was plotted as a function of time. The dissolution profiles of APR with standard errors were shown in Fig. 16. Three repeated experiments were completed and the standard errors distributed in the range of 1.132%–8.465%. It is seen from Fig. 16 that unprocessed APR and physical mixture of PVP/APR 3:1 achieve about 60% and 63% release in 2 h, whereas the SAS processed APR reaches 80% dissolution in 2 h which is due primarily to the particle size reduction and interfacial surface area increase. The coprecipitate of PVP/APR 3:1 and 9:1 processed at 12 MPa, 40 °C, in which microparticles were formed, show radically different behaviors, with dissolution profile in which about 100% of the drug were released in 60 min and 30 min, respectively. Moreover, the similarity factor (*f*_*2*_) was calculated to evaluate a dissolution of an active pharmaceutical ingredient in the light of FDA and European Medicines Agency. Therefore, the *f*_*2*_ obtained of the SAS processed APR, PVP/APR 3:1, PVP/APR 9:1 were 43, 18 and 13 respectively, which were less than 50, indicating that the dissolution curves were dissimilar.

Since DMF belongs to Class 2 in the FDA guidance, the maximum acceptable concentration of DMF in the final product is 880 ppm^36,37^. The solvent residue content in PVP/ARP microcapsules was measured by a GC-FID. The solvent residue was 676 ppm when the drying time was 60 min, indicating that it was in the acceptable range of FDA guidance.

## Discussion

In order to interpret the results obtained, it is necessary to recall the general SAS precipitation mechanisms. The formation of the particle morphology is governed by two characteristic times: the time of jet breakup (t_JB_) and the time of surface tension vanishing (t_STV_)^38–40^. When SAS is performed under the MCP, microparticles are obtained by the liquid jet breakup, micrometric droplet formation and drying, as a consequence of the atomization process. When SAS is performed above the MCP, single-phase mixing like “gas-plume” is the only fluid dynamic behavior, and interfacial tension disappears before jet break-up. The precipitation mechanism is governed by gas-to-particle nucleation, therefore, nanoparticles formed directly from the solute. Applying this interpretation to the microcapsules, if t_JB_ is the shorter characteristic time, composite polymer/drug microparticles can be formed, since the two compounds are entrapped in the droplet that behaves like a confined reactor; on the contrary, if t_STV_ is the shorter characteristic time, the polymer and the active compound precipitate separately by different nucleation and growth times, thus, the microcapsules fails^33^.

Dissolution tests confirmed these conclusions, since SAS processed microcapsules of PVP/APR 3:1 and 9:1 exhibited a dramatic reduction of the time required to complete 100% dissolution, with respect to the unprocessed samples. In this case, the dissolution of APR was improved significantly. The similar trends were drawn by^41,42^. This may be ascribed to the fact that APR is dispersed in the form of nanoparticles inside the PVP matrix^43^. Meanwhile, PVP may improve the surface properties of the particles and enhance the wettability due to its wetting effect; hence, the drug dissolution was improved.

Besides, the effect of process parameters on particle morphology and mean size were discussed to achieve the further goal. As known, polymer/drug ratio is the crucial factor influencing particle morphology. When the polymer content of the solution increases, the viscosity of solution increases, resulting in polymer chain entanglement; meanwhile, the increase of solution viscosity may reduce the diffusion rate of CO_2_ into the solution and hinder the mass transfer between CO_2_ and solution, thus, larger particles produced^43^. The study on the effect of pressure showed that, increasing the operating pressure (thereby, increasing the density of CO_2_), the particle mean size decreased and the PSD shrank. As already reported by Sarah et al.^44^, increasing the pressure, the density difference between the CO_2_ and solvent reduced; as a result, the mass transfer and supersaturation enhanced, leading to the faster nucleation and smaller particle size. However, microparticles obtained at 15 MPa is unusual, since, the operating points of these conditions may be located far above the MCP of the CO_2_/DMF binary system, and, therefore, nanoparticles precipitation could be occurred^45^. This behaviour can be interpreted by hypothesizing that the presence of PVP maintains this morphology in an extensive range of pressures in SAS. Varying the temperature, it was possible to note that, increasing the temperature, the average diameter increased and the PSD enlarged. From the perspective of phase equilibrium, the MCP of the CO_2_/DMF binary system shifts towards higher pressures with temperature increasing, thus, the distance between the operating points and the MCP reduced and larger particles were obtained; on the other hand, from the perspective of thermodynamic, higher temperature increases the solubility of the solute in a solvent and reduces the CO_2_ power solvent, resulting in lower supersaturation and larger particles^45,46^. It can be noted that the particle size increase slightly with the overall solute concentration. An increase of the total concentation produces an increase of the solution viscosity; on the other hand, enough solute molecules promote the nucleus of microparticle growing in high total concentation conditions, therefore, the growth process of nucleus become the main mechanism, with consequent formation of larger particles.

The drug powder collected every hour is in the kilogram level. Considering that the possibility of scaling up the production of pharmaceuticals via SAS technology has been achieved, from an industrial perspective, the satisfying results of this study are very promising.

## Conclusions

In this work, it was demonstrated that it is possible to obtain the successful APR microcapsules by SAS using PVP as the carrier for the first time. Mean particle size increased with PVP/APR ratio and temperature, decreased with pressure and changed insignificantly with overall solute concentration. Dissolution tests verified that the drug release of the PVP/APR coprecipitate is enhanced greatly compared with unprocessed APR. The results obtained in this study are promising as it verified the possibility to scale up the production of pharmaceuticals employing the SAS process from an industrial point of view.

## Data Availability

The datasets used and/or analysed during the current study available from the corresponding author on reasonable request.
